# Reduction of Benzodiazepine Use in Patients Prescribed Medical Cannabis

**DOI:** 10.1089/can.2018.0020

**Published:** 2019-09-23

**Authors:** Chad Purcell, Andrew Davis, Nico Moolman, S. Mark Taylor

**Affiliations:** ^1^ Dalhousie Medical School Class of 2019, Dalhousie University Faculty of Medicine, Undergraduate Medical Education, Halifax, Canada.; ^2^ Department of Economics, Acadia University, Wolfville, Canada.; ^3^ Division of Otolaryngology—Head and Neck Surgery, Department of Surgery, Dalhousie University, Halifax, Canada.

**Keywords:** benzodiazepines, deprescribing, discontinuation, medical cannabis

## Abstract

**Background:** Benzodiazepines are a class of medication with sedative properties, commonly used for anxiety and other neurological conditions. These medications are associated with several well-known adverse effects. This observational study aims to investigate the reduction of benzodiazepine use in patients using prescribed medical cannabis.

**Methods:** A retrospective analysis was performed on a cohort of 146 medical cannabis patients (average age 47 years, 61% female, 54% reporting prior use of cannabis) who reported benzodiazepine use at initiation of cannabis therapy. These data are a part of a database gathered by a medical cannabis clinic (Canabo Medical). Descriptive statistics were used to quantify associations of the proportion of benzodiazepine use with time on medical cannabis therapy.

**Results:** After completing an average 2-month prescription course of medical cannabis, 30.1% of patients had discontinued benzodiazepines. At a follow-up after two prescriptions, 65 total patients (44.5%) had discontinued benzodiazepines. At the final follow-up period after three medical cannabis prescription courses, 66 total patients (45.2%) had discontinued benzodiazepine use, showing a stable cessation rate over an average of 6 months.

**Conclusion:** Within a cohort of 146 patients initiated on medical cannabis therapy, 45.2% patients successfully discontinued their pre-existing benzodiazepine therapy. This observation merits further investigation into the risks and benefits of the therapeutic use of medical cannabis and its role relating to benzodiazepine use.

## Introduction

Benzodiazepines are a class of medications commonly used to treat a variety of neurological conditions.^1^ Hypnotic and anxiolytic properties make benzodiazepines a mainstay in the treatment of insomnia and anxiety disorders, as well as alcohol, seizure, and spasticity disorders. These effects are exerted by amplification of inhibitory neural signaling, primarily via gamma-aminobutyric acid receptors.^2^ A comprehensive review of the pharmacologic properties of benzodiazepines is outside the scope of this study, but can be found elsewhere.^3^

Annual incidence rates of benzodiazepine use vary across North American populations and regions, with estimates upward of 10%.^4,5^ Likewise, Canadian survey data suggest benzodiazepine use has consistently been within the range of 5% in 2003 to 10% in 2012.^6^ Benzodiazepines are considered to have a relatively good safety profile in comparison with older sedative hypnotics, such as barbiturates. However, common side effects include ataxia, dizziness, drowsiness, fatigue, slowed reaction, and muscle weakness.^1^ Complications of long-term use include lack of concentration, dependence, tolerance, overdose, and addiction.^2^ A recent meta-analysis found increased mortality in benzodiazepine users compared with nonusers, with a hazard ratio (HR) of 1.6 (*p*≤0.05).^7^ This has similarly been shown in a systematic review demonstrating an increase in overall mortality in regular benzodiazepine users, with a HR ranging from 1.2 to 1.7 in the studies reviewed.^8^ While benzodiazepines remain an essential class of medication, there is certainly need for caution regarding its side effect profile.

This study seeks to investigate benzodiazepine discontinuation rates in a population of patients referred for medical cannabis therapy.

## Materials and Methods

A retrospective analysis was performed on a cohort of patients using medical cannabis. These data are part of an ongoing database gathered by Canabo Medical Clinic on medical cannabis patients. At the time of this study, there were 10 clinics operating in Ontario, Alberta, Nova Scotia, and Newfoundland, accepting patients exclusively through referral. Canabo clinics comprised physicians who specialize in the controlled prescribing of medical cannabis for a variety of medical conditions, to whom other health care providers can refer patients. All patients in the study were referred to Canabo by practicing physicians outside the clinic network. Deidentified patient data were obtained in collaboration with Canabo Medical and the electronic health records provider, InputHealth.

Canabo physicians collect self-reported patient information at each clinic visit. Three follow-up appointments were considered adequate to ensure sufficient collection of patient-reported information. Physicians typically wrote prescriptions for 2-month periods, with an average period between visits of 61.3 days, although exact times varied with patient schedules and physician discretion. Based on average prescription durations, patients could reach three follow-up visits in just over 6 months. Due to variability in prescribing practices and adherence, patients enrolled within 9 months of the study end date could be included. The study end date was October 31, 2016, and patients were eligible for inclusion if their first visit to Canabo was before January 31, 2016. Canabo records identified 884 patients who were using benzodiazepines at the time of their initial visit to the clinic, before prescription of cannabis. Six hundred seventy-seven patients were excluded because sufficient information could not be collected before the study end date. Of the 207 patients who initiated cannabis before January 31, 2016, 146 (70.5%) completed three follow-up visits and formed the study sample. No patients were excluded for any other reason, including past medical cannabis usage, indication for medical cannabis or benzodiazepines, previous discontinuation, or any observable characteristics. To evaluate patient's perceived burden of disease, they were asked “how often is your life affected/impacted by your medical condition,” and given options to answer were as follows: “all the time,” “most of the time,” and “occasionally/rarely” (Table 2).

Benzodiazepine management and discontinuation was not a specific goal of any Canabo physician, and benzodiazepine cessation may have been initiated by a physician or patients. Patients were not tested for verification of reported benzodiazepine discontinuation. Referring physicians were sent consultation and follow-up notes regarding their respective patient's progress under medical cannabis treatment.

Statistical testing via binomial *t*-tests was used to assess population mean differences in benzodiazepine use after each clinic visit, following initiation of medical cannabis therapy. Approaches involving estimating regression models were deemed unsuitable, given sample size limitations. The potential relevance of the Δ9-tetrahydrocannabinol (THC) and cannabidiol (CBD) content of cannabis used, and differences in the patients' perceived impact of their medical condition(s) on their life were evaluated using chi-square tests.

## Results

Sample demographics did not identify any significant discrepancies between patients who discontinued their benzodiazepines and those who did not. The mean age of participants was 47.7 years with a standard deviation of 12.7 years. Prior use of cannabis was self-reported by 54% of patients. A total of 97.6% of patients were not currently using other recreational drugs and 73.3% had never used recreational drugs that were not cannabis. Concurrent alcohol and cigarette use was reported in 41.4% and 30.8% of patients, respectively. Results on the discontinuation of benzodiazepine use by each of these groups are presented in Table 1.

**Table 1.** Sample Demographics and Outcomes

	Mean	Final BD use	Final no BD use	Significance of difference
Age (years)	47.7	47.2	48.3	NS (*p*>0.1)
Female	61.0%	61.3%	60.6%	NS (*p*>0.1)
Using cannabis at intake	54.3%	50.3%	59.1%	NS (*p*>0.1)
No current recreational drug use	97.6%	98.1%	97.0%	NS (*p*>0.1)
No history of non-cannabis recreational drug use	73.0%	73.1%	72.7%	NS (*p*>0.1)
Current alcohol use	41.4%	41.9%	40.9%	NS (*p*>0.1)
Current cigarette use	30.8%	32.5%	28.8%	NS (*p*>0.1)
Chronic condition (>3 years)	80.1%	80.9%	80.0%	NS (*p*>0.1)

BD, benzodiazepine; NS, not significant.

Reported primary conditions driving cannabinoid treatment were grouped into neurological (7.5%), pain (47.9%), psychiatric conditions (31.9%), and other (12.7%). Small sample sizes prevent a strong assessment of the link between medical condition and benzodiazepine use.

**FIG. 1.**
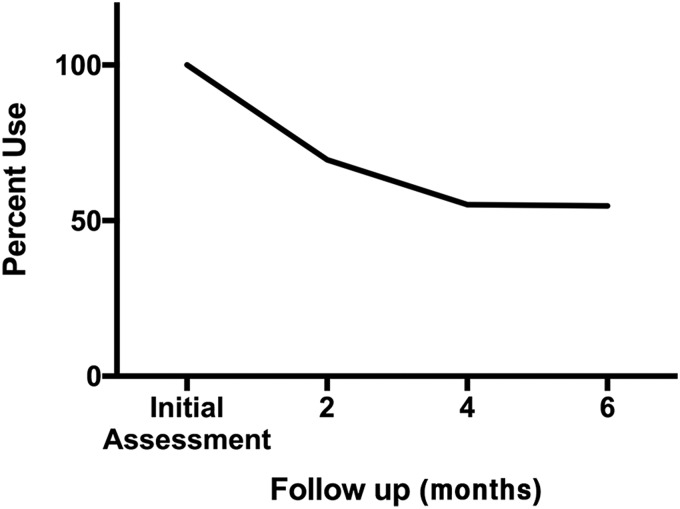
Percent decline in benzodiazepine use among patients at follow-up time points after initiating medical cannabis treatment.

After the first visit, 44 patients (30.1%) had discontinued their benzodiazepines. Another 21 patients, for a total of 65 patients (44.5%), had discontinued their benzodiazepines by the second visit. By the third visit, one more patient had discontinued benzodiazepines, for a total of 66 patients (45.2%) The reduction between the initial visit and first follow-up is significant at the *p*<0.001 level, as is the reduction between the first follow-up and the second and third follow-up visits (Fig. 1).

On their initial visit, 74% of patients reported that their “life is affected/impacted by [my] medical condition” “all the time.” After three clinic visits, 45.0% of patients using benzodiazepines, and 30.3% of patients who discontinued benzodiazepines reported that their life was impacted by their medical condition “all the time” (Table 2; chi-square >0.1).

**Table 2.** Self-Reported Impact of Medical Condition on Quality of Life by All Patients at Initial Visit to Canabo Clinic, and by Benzodiazepine Use Status After Three Visits

	Impact on life	First visit—all patients	After three visits—BD use	After three visits—no BD use
How often is your life affected/impacted by your medical condition	All the time	74.0%	45.0%	30.3%
	Most of the time	22.6%	38.8%	43.9%
	Occasionally/rarely	3.4%	16.3%	25.8%
				Chi-square >0.1

The distribution of cannabinoid (CBD and THC) proportions was not significantly different among patients who continued and those who discontinued benzodiazepines (Table 3; chi-square >0.1).

**Table 3.** Δ9-Tetrahydrocannabinol and Cannabidiol Content of Cannabis Used by a Sample Population by Benzodiazepine Use Status

	Mean	Final BD use	Final no BD use
THC level			
>20%	20.2%	14.7%	26.8%
15–19.9%	34.7%	36.8%	32.1%
10–14.9%	12.1%	16.2%	7.1%
5–9.9%	13.7%	14.7%	12.5%
1–4.9%	7.3%	7.4%	7.2%
<1%	4.0%	2.9%	5.4%
Unknown	8.1%	7.4%	8.9%
			Chi-square >0.1
CBD level			
>17%	7.3%	5.9%	8.9%
12–16.9%%	13.7%	14.7%	12.5%
8–11.9%	18.6%	14.7%	23.2%
4–7.9%	13.7%	16.2%	10.7%
1–3.9%	8.1%	11.8%	3.6%
<1%	17.8%	14.7%	21.4%
Unknown	21.0%	22.1%	19.7%
			Chi-square >0.1

CBD, cannabidiol; THC, Δ9-tetrahydrocannabinol.

## Discussion

Patients initiated on medical cannabis therapy showed significant benzodiazepine discontinuation rates after their first follow-up visit to their medical cannabis prescriber, and continued to show significant discontinuation rates thereafter. Discontinuation was not associated with any measured demographic characteristic. Patients also reported decreased daily distress due to their medical condition(s) following prescription cannabinoids. The CBD and THC content of cannabis used did not differ among patients who continued and those who discontinued benzodiazepines.

The observed association between medical cannabis use and benzodiazepine discontinuation should not be misinterpreted as causative, and these results do not support inferences about substitution of medical cannabis for benzodiazepine therapy. Substitution of cannabis for opioids is supported by a growing body of evidence, although many challenges and unknowns limit widespread adoption of cannabis use for this indication.^9,10^ Extrapolation of self-reported data suggests that there are potentially as many Canadians using cannabis for its sedative and anxiolytic properties as there are patients taking sedatives such as benzodiazepines.^11,12^ The substitution effect of medical cannabis has also been seen with medications for pain, anxiety, migraine, depression, chronic pain, and headache.^10,13,14^

This study found no significant difference in the proportions of CBD and THC in the cannabis used by patients who continued and those who discontinued benzodiazepines. This study did not intend to address the relationship between cannabis and anxiety, or the physiological mechanisms of THC and CBD. However, review of the literature on this topic is warranted, as the effects of cannabis on anxiety are not fully understood.^15^ Animal studies have reported anxiolytic effects of whole cannabis administration.^16^ Experimental animal evidence cannot be easily generalized to human consumption because the use of dried cannabis flower may exhibit varying effects. These effects may be dependent on factors such as proportions and interactions among cannabinoids, amount used, and method of use. The potency of cannabis has been increasing over the past two decades. THC content has been increasing and CBD content decreasing, resulting in an increase in THC:CBD ratios from 14:1 in 1995 to 80:1 in 2014.^17^ Previous studies have reported associations of high THC/low CBD content with increased risk of anxiety.^18^ CBD and THC have proposed conflicting effects on anxiety. CBD has been associated with anxiolytic effects regardless of dose, while THC reliably produces subjective effects of anxiety, but appears to be anxiolytic at lower doses and anxiogenic at higher doses.^15,19^ Pre-clinical studies of CBD have demonstrated promise in treating anxiety disorders.^20^ Animal models support a reduction of anxiety symptoms in relation to generalized anxiety disorder and post-traumatic stress disorder through CBD treatment.^21,22^ Human and animal studies suggest that CBD may have role in attenuating the effects of THC, including anxiety.^15^ The observed association of benzodiazepine discontinuation with use of medical cannabis highlights the importance of further characterization of the anxiolytic properties of cannabis in the future.

Medical cannabis use has increased dramatically in recent years. The total number of Canadians registered for medical cannabis as of September 30th increased from 12,409 in 2014, to 30,537 in 2015 and 98,460 in 2016.^23^ Canabo clinics experienced similar growth from 2014 through 2015 with patient volumes expanding by more than double each year. Canabo data accessed in October 2016 consisted primarily of new patients from the same calendar year. Of the 207 patients who initiated medical cannabis with Canabo by January 31, 2016, 61 patients did not complete three visits before study end date. The designation of a 9-month window to receive three follow-up visits may contribute to these lost 61 patients. These patients may have received prescriptions for >3 months, or used their medical cannabis less frequently than discussed with their physician. Patient reasons for discontinuing clinic treatment are unknown and may include the typical reasons for loss to follow-up from any medical clinic.

There are several limitations to the current study. This study is not designed to, nor should be used to hypothesize physiological mechanisms to explain this observed association between benzodiazepines and cannabis. The retrospective observational methodology and sample size preclude an inference of a causal relationship between cannabis and benzodiazepine use trends. Sample size limitations also preclude our ability to make inference from the smaller proportion of benzodiazepine discontinuers than continuers who reported that their medical condition affected their life all the time after three clinic visits. Without dependable safety data and evidence from randomized trials for this cohort, cannabis cannot be recommended as an alternative to benzodiazepine therapy. Retrospective analysis of pre-existing Canabo data from ongoing clinic standard operating procedures precludes examination of many potentially valuable parameters for study, such as benzodiazepine dosing, indication and duration of use, and information about patients' intentions with discontinuation. No objective measure of benzodiazepine discontinuation was used to confirm self-reported data. Future studies could make use of biomarkers to more closely characterize benzodiazepine discontinuation. Although relative proportions of THC and CBD were reported, data did not include the strain of cannabis, or method of use. Consistent use of cannabis was approximated by patients returning to clinic three times after initial visit. Consistent use was thereby inferred from patients consistently returning to clinic. The present study demonstrates an association between medical cannabis therapy and reductions in benzodiazepine use. There is a fundamental paucity of research on the effectiveness of cannabis as a medical therapy, as well as the risks and benefits of its use.^24^ Future studies should aim to expand on the current understanding of cannabis and its potential medical applications.

## Conclusion

Medical cannabis remains a controversial but potentially effective treatment for patients suffering from a variety of medical conditions. Within a cohort of patients initiated on medical cannabis therapy, a large proportion successfully discontinued their pre-existing benzodiazepine therapy. This study therefore supports the continued research of medical cannabis and urges further exploration into its therapeutic value.

## Abbreviations Used

CBD: cannabidiol
HR: hazard ratio
THC: Δ9-tetrahydrocannabinol
